# Homelessness and Psychosis: Psychiatric Diagnoses and Comorbidities in a Portuguese Cohort

**DOI:** 10.1192/j.eurpsy.2026.10471

**Published:** 2026-07-31

**Authors:** J. Gama-Marques, J. Henriques-Calado

**Affiliations:** 1 Centro de Investigação em Ciência Psicológica (CICPSI), Faculdade de Psicologia, Universidade de Lisboa, Lisboa, Portugal; 2 Consulta de Esquizofrenia Resistente, Hospital Júlio de Matos, Unidade Local de Saúde São José, Centro Clínico Académico de Lisboa, Lisboa, Portugal; 3 Clínica Universitária de Psiquiatria e Psicologia Médica, Faculdade de Medicina, Universidade de Lisboa, Centro Académico de Medicina de Lisboa, Lisboa, Portugal

## Abstract

**Introduction:**

The prevalence of psychotic disorders is significantly associated with homelessness. The condition of homelessness exacerbates common psychotic disorders, resulting in treatment non-compliance, increased substance abuse, and decreased survival rates. Despite its public health importance, long-term studies on homelessness and psychosis are scarce.

**Objectives:**

This research sought to outline the socio-demographic, diagnostic and clinical features of homeless psychiatric patients with psychotic disorders in Portugal.

**Methods:**

A retrospective analysis was conducted on 295 homeless psychiatric patients diagnosed with psychotic disorders, based on electronic clinical records. ICD-10 diagnoses, sociodemographic, and clinical data were recorded. Descriptive statistics and inferential analyses were computed.

**Results:**

The cohort was predominantly male (78.3%), with a mean age of 41.7 years at initial contact. Mean psychiatric follow-up lasted 6 years, encompassing 3.26 admissions and 148 inpatient days. Unspecified nonorganic psychosis (ICD-10 F29; 44.7%) and organic psychosis (ICD-10 F06; 37.6%) were the most frequent diagnoses, while schizophrenia (ICD-10 F20; 11.2%) and schizoaffective disorders (ICD-10 F25; 6.5%) were less common. Personality disorders were more frequent among younger patients (*p*<.01). Substance use was highly prevalent, with alcohol abuse in 51.5% and cannabis in 40.7% of cases, alongside significant cocaine (6.4%) and heroin (4.7%) use. Gender associations were pronounced: men displayed significantly higher alcohol (χ²=18.52, *p*<.001) and cannabis use (χ²=9.97, *p*<.001) compared with women. Age strongly influenced diagnostic patterns: older patients were more likely to present with organic psychosis (*M*=50.5 years; *p*<.001), whereas younger individuals showed increased cannabis (*M*=44.5 years; *p*<.001) and cocaine use (*M*=44.0 years; *p*<.001).

**Conclusions:**

Homeless psychiatric patients with psychotic disorders exhibit a distinctive profile, marked by a high prevalence of organic and unspecified psychoses, substantial comorbidity related to substance use, and significant age- and gender-related diagnostic variations. The findings emphasize the complexity of psychotic disorders in real-world homelessness clinical settings and reinforce the need for tailored psychiatric, and substance use interventions throughout lifespan within homeless services.

**Disclosure of Interest:**

None Declared

